# Critical Period of Nonpromoter DNA Methylation Acquisition during Prenatal Male Germ Cell Development

**DOI:** 10.1371/journal.pone.0024156

**Published:** 2011-09-19

**Authors:** Kirsten M. Niles, Donovan Chan, Sophie La Salle, Christopher C. Oakes, Jacquetta M. Trasler

**Affiliations:** Departments of Pharmacology and Therapeutics, Pediatrics and Human Genetics, McGill University and Research Institute at The Montreal Children's Hospital of the McGill University Health Centre, Montréal, Quebec, Canada; CNRS, France

## Abstract

The prenatal period of germ cell development is a key time of epigenetic programming in the male, a window of development that has been shown to be influenced by maternal factors such as dietary methyl donor supply. DNA methylation occurring outside of promoter regions differs significantly between sperm and somatic tissues and has recently been linked with the regulation of gene expression during development as well as successful germline development. We examined DNA methylation at nonpromoter, intergenic sequences in purified prenatal and postnatal germ cells isolated from wildtype mice and mice deficient in the DNA methyltransferase cofactor DNMT3L. Erasure of the parental DNA methylation pattern occurred by 13.5 days post coitum (dpc) with the exception of approximately 8% of loci demonstrating incomplete erasure. For most loci, DNA methylation acquisition occurred between embryonic day 13.5 to 16.5 indicating that the key phase of epigenetic pattern establishment for intergenic sequences in male germ cells occurs prior to birth. In DNMT3L-deficient germ cells at 16.5 dpc, average DNA methylation levels were low, about 30% of wildtype levels; however, by postnatal day 6, about half of the DNMT3L deficiency-specific hypomethylated loci had acquired normal methylation levels. Those loci normally methylated earliest in the prenatal period were the least affected in the DNMT3L-deficient mice, suggesting that some loci may be more susceptible than others to perturbations occurring prenatally. These results indicate that the critical period of DNA methylation programming of nonpromoter, intergenic sequences occurs in male germline progenitor cells in the prenatal period, a time when external perturbations of epigenetic patterns could result in diminished fertility.

## Introduction

DNA methylation is an epigenetic modification that affects transcriptional silencing, X-chromosome inactivation, and genomic imprinting without changing the underlying genetic code. Catalyzed by the DNA methyltransferase (DNMT) enzymes, DNA methylation patterns are first established in a precise manner during gametogenesis. Upon primordial germ cell (PGC) migration into the primitive mouse gonad at 10.5 dpc, nearly complete erasure of the parental somatic cell DNA methylation pattern occurs genome-wide as well as at imprinted and single-copy genes [1], [2], [3], [4]. Repetitive elements such as LINE1, IAP, SINEB1 and minor satellites undergo incomplete demethylation [1], [2], [4], [5], [6], [7]. Erasure of the parental pattern in the germline is thought to be essential for resetting DNA methylation both to ensure gender-specific methylation of imprinted genes as well as to prevent transgenerational inheritance of abnormal DNA methylation patterns. Evidence for transgenerational inheritance through the germline has been reported in both the Agouti (*A^vy^*) and *Axin^Fu^* mouse models [8], [9] indicating that some loci might escape erasure in PGCs and have adverse consequences for the offspring.

Following erasure, DNA methylation is acquired in a gender-specific manner from ∼14.0 dpc until shortly after birth at the paternally methylated imprinted genes *H19*, *Rasgrf1*, and *Dlk1/Gtl2* in male germ cells [1], [10], [11]. *H19* undergoes allele-specific timing of DNA methylation with complete methylation establishment on the paternal allele prior to birth and on the maternal allele by the pachynema in spermatocytes [12], [13]. Repetitive elements complete DNA methylation acquisition prior to birth [1], [7]. In our previous studies, examination of DNA methylation at many sites across the genome in postnatal spermatogenesis revealed that the vast majority of methylation acquisition in male germ cells is completed prior to the formation of type A spermatogonia that are present at day 6 after birth. Changes in DNA methylation continued to occur at a small number of sites until germ cells reached pachynema [14]. Many methylation differences identified between sperm and somatic tissues in the mouse, however, were found to be located at nonpromoter, intergenic, non-repetitive sequences rather than at genes critical in germ line development [15]. Recently, genome-wide approaches, including next generation sequencing, in mouse and human have demonstrated that DNA methylation is frequently found in regions outside of proximal promoters, including intergenic sequences and gene bodies, and appears to play an important role in regulating developmental gene expression [16], [17].

While many tissues demonstrate tissue-specific DNA methylation, patterns of DNA methylation in mature spermatozoa from both mouse and human are distinctly unique from those in somatic tissues [15], [18], [19]. Sperm DNA methylation patterns may play important functional roles in spermatogenesis or the resulting embryo. The germline has a unique global transcriptional profile due to the large number of genes necessary for meiosis and spermatogenesis [20], and it has been suggested that DNA methylation may contribute to the control of gene expression programs essential for successful gametogenesis [21]. In addition, recent studies suggest that epigenetic modifications including those on histones retained in sperm and DNA methylation, may have functional consequences for the offspring and contribute to the regulation of gene transcription in the embryo post-fertilization [19], [21], [22].

DNMT mouse mutants indicate that correct acquisition of DNA methylation patterns in the male germ line is critical for spermatogenesis. Germline deficiency of DNMT3a [23], involved predominantly in *de novo* methylation during gametogenesis [24], [25], and DNMT3L [26], [27], an enzyme with no intrinsic methyltransferase activity [28] but that cooperates with DNMT3a in *de novo* methylation [29], both result in azoospermia and infertility [25], [30], [31], [32]. Aberrant methylation of paternally methylated imprinted genes and repetitive DNA elements has been observed in both the DNMT3a and DNMT3L deficiency models in prospermatogonia, type A spermatogonia, and pre-meiotic spermatocytes [1], [25], [31], [33]. Additionally, methylation analysis of intergenic sequences on chromosomes 4 and X demonstrated a loss of methylation in postnatal Type A spermatogonia in the absence of DNMT3L as well as a decrease in germ cell number by day 6 [34]. The ontogeny of the methylation of intergenic sequences during the prenatal period of acquisition, following erasure in primordial germs cells, has not been examined.

Although cell numbers are limiting, a number of studies have described the acquisition of DNA methylation at some sites within unique sequences, imprinted regions and repeat sequences during male germline development, beginning in the prenatal period. Other studies have indicated that prenatal male germ cells may be sensitive to *in utero* maternal exposures to environmental toxins such as Bisphenol A as well as methyl donor deficiency [35]. In addition, maternal supplementation with the alternative methyl donor betaine improved the testicular histology of methylenetetrahydrofolate-reductase (MTHFR) - deficient male offspring [35]. Taken together, these studies indicate that prenatal germ cell development is a key period for DNA methylation acquisition and as such male germ cells may be exquisitely sensitive to maternal exposures such as environmental toxicants and dietary restrictions. With the recent identification of an important role for nonpromoter sequence methylation in gene regulation during development [17], our goal here was to examine the extent of erasure and subsequent dynamics of intergenic DNA methylation pattern acquisition during prenatal and early postnatal male germline development in both normal and DNMT3L deficient mice.

## Results

### Intergenic sequences have a unique pattern of DNA methylation in sperm

**Figure 1:**
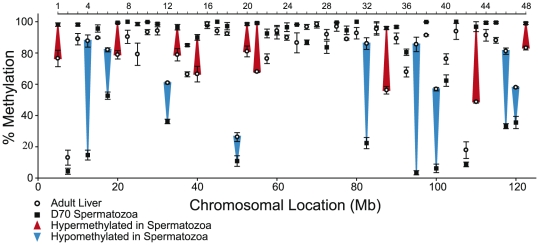
qAMP analysis of DNA methylation at intergenic sites along chromosome 9 in day 70 liver (n = 3) and spermatozoa (n = 3). Nine sites showed hypermethylation and nine sites showed hypomethylation in spermatozoa compared to liver demonstrating the unique pattern of DNA methylation present in mature sperm. Error bars represent standard error of the means (SEM).

DNA methylation analysis using restriction landmark genomic scanning (RLGS) in mice determined that the sperm DNA methylation pattern differed significantly from that of somatic tissues examined especially at non-repetitive, non-CpG island intergenic sequences [15]. Confirmation of this finding was obtained by examining the DNA of mature spermatozoa and liver using a chromosome based approach that analyzed intergenic sites spanning chromosomes 4, 10, 17 and X with quantitative analysis of DNA methylation by real-time PCR (qAMP) [15], [36]. A subsequent developmental study showed that intergenic sequences on chromosomes 4, 7, 10, 17 and X acquire most of their methylation prior to the Type A spermatogonia phase of development; the results suggested that the prenatal period might be an important time for the methylation of intergenic sequences, as had been reported for imprinted and repeat sequences [14]. Here, we first extended our previous analysis of intergenic sites on chromosomes 4, 7,10, 17 and X to include chromosome 9 and increased the resolution of methylation analysis by examining twice as many sites along this chromosome. Similar to our previous studies, intergenic sites on chromosome 9 were chosen that were not proximal (>10 kb away) to CpG islands or the transcriptional start site of a known gene and were in non-repetitive regions [14], [15]. Forty-eight intergenic regions spanning chromosome 9 at 2.5 Mb intervals were selected and primers were designed for qAMP. DNA methylation levels were first examined at the selected regions in day 70 spermatozoa and liver isolated from three adult males. Results showed that in spermatozoa as compared to liver tissue, nine sites were hypomethylated and nine sites were hypermethylated (Fig. 1) further confirming the unique patterns of DNA methylation present in male germ cells. While most of the intergenic sites showed high levels of methylation (80–100% methylation), about 12/48 sites showed low (<20% methylation) or moderate (20–80%) levels. As the germ cell-specific patterns of intergenic methylation along chromosome 9 were similar to what had been described for other chromosomes [15], these sequences were used here to determine the developmental timing of intergenic sequence methylation in developing male germ cells.

### Erasure of DNA methylation at intergenic sites in primordial germ cells

**Figure 2:**
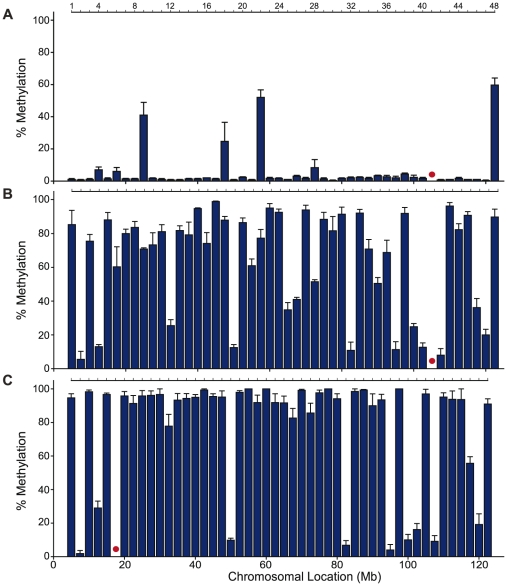
qAMP analysis of DNA methylation along chromosome 9 in male germ cells from A) 13.5 dpc embryos (only HhaI digests are shown, n = 3), B) 16.5 dpc (n = 3) embryos, and C) day 6 (n = 3) mice show that the critical period of DNA methylation establishment for intergenic sequences occurs during prenatal germ line development. Red circles indicate no data available. Error bars represent SEM.

**Figure 3:**
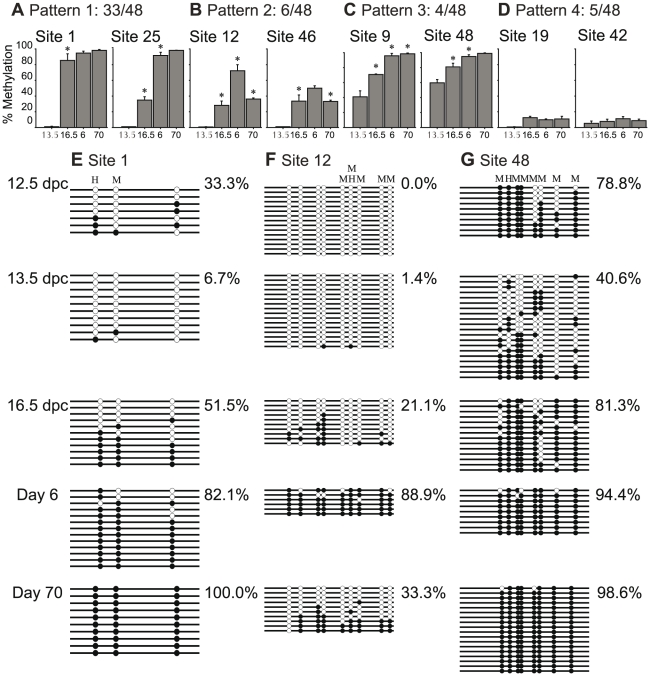
Examination of individual intergenic sites revealed that methylation acquisition occurs dynamically throughout germ cell development resulting in four different patterns. Results from only HhaI digestions are shown for all 13.5 dpc data (n = 3 for each time point). Error bars represent SEM. Asterisks denote a significant (t-test, p<0.05) change in DNA methylation compared to the previous time point. **A)–D)** Representative sites for each of the four observed patterns at 13.5 dpc, 16.5 dpc, day 6 and day 70; **E)–G)** Confirmation of the DNA methylation patterns determined by qAMP was completed using bisulfite sequencing of sites 1, 12 and 48 at 13.5 dpc, 16.5 dpc, day 6, and day 70. In addition, bisulfite sequencing of the same sites at 12.5 dpc suggested that erasure is only completed by 13.5 dpc.

The erasure of somatic DNA methylation patterns in PGCs at imprinted and unique gene loci has been found to be nearly complete while repetitive DNA elements retain higher levels [1], [7], [10], [11], [12], [13], [37]. Occasional evidence of approximately 10% residual DNA methylation at imprinted and unique gene loci has frequently but not in all cases been attributed to somatic cell contamination [1], [11], [13]. To determine the methylation erasure status at intergenic sequences, GFP-positive 12.5 dpc and 13.5 dpc male germ cells obtained from timed matings between Oct4-GFP male mice [38] and CD1 females were collected by flow cytometry. First, DNA methylation at 48 selected intergenic sites along chromosome 9 was analyzed in three groups of pooled germ cells isolated from 13.5 dpc fetuses using qAMP. Results from only the HhaI digestions were examined since the McrBc enzyme functions poorly at low levels of DNA methylation [36]. DNA methylation at 13.5 dpc was low (average 5.52%) showing complete erasure at 43/48 sites coinciding with the erasure phase in PGCs (Fig. 2A). Sites 9, 18, 22, and 48 were exceptions demonstrating DNA methylation levels of 41%, 25%, 52%, and 60% at the HhaI restriction site respectively (Fig. 2A). Bisulfite sequencing at site 48 was performed on DNA isolated from 12.5 dpc and 13.5 dpc gonocytes to ensure that germ cells were examined at a sufficiently early time point and to confirm the qAMP results. Overall DNA methylation at 12.5 dpc and 13.5 dpc was 78.8% and 40.6% respectively demonstrating the continual erasure of DNA methylation in PGCs from 12.5 dpc to 13.5 dpc as well as confirming the absence of complete erasure determined in the qAMP analysis (Fig. 3G). Incomplete erasure of methylation was also found at intergenic sites on other chromosomes. On chromosome 4, a similar small number of sites, two out of 27 (<10%), showed methylation values greater than 50% at 13.5 dpc (Fig. S1).

### A major period of DNA methylation acquisition at intergenic sites in male germ cells occurs during prenatal gametogenesis in a site-specific manner

In male germ cells, DNA methylation acquisition commences at approximately 14–15.5 dpc at unique gene loci, imprinted genes, and repetitive elements [1], [5], [7], [10], [11], [12], [37]. RLGS studies indicated that the majority of DNA methylation acquisition occurs in prospermatogonia prior to the type A spermatogonia stage present at day 6, with a small number of genomic regions continuing modification until the pachytene spermatocyte phase [14]. Since the prenatal and early postnatal dynamics of DNA methylation acquisition at intergenic sites are not known, pure populations of germ cells were isolated by flow cytometry from GFP-positive males at 16.5 dpc and day 6 from timed matings between CD1 females and GFP-positive males and DNA methylation at the 48 selected intergenic regions spanning chromosome 9 was analyzed with qAMP at both time points. At 16.5 dpc, the average DNA methylation across chromosome 9 had increased to 62.0%, a significant (p<0.001) increase from 13.5 dpc (Fig. 2B). From 16.5 dpc to day 6, the average of DNA methylation on chromosome 9 significantly increased (p = 0.003) to 77.5% (Fig. 2C). This period of acquisition coordinates well with the timing of increased expression from 15.5 dpc of the *de novo* methyltransferase enzymes, Dnmt3a2 and Dnmt3L, that cooperate in prenatal germ cell DNA methylation acquisition (Fig. S2, S3).

Examination of DNA methylation at individual sites during the time period investigated revealed four main patterns of acquisition dynamics (Fig. 3A–D). The majority of sites (33/48) demonstrated an increase in DNA methylation at each subsequent time point attaining the highest levels of methylation at the end of their acquisition phase (Fig. 3A). The second pattern present at 6/48 sites initially demonstrated an increase in DNA methylation (Fig. 3B). Upon completion of methylation acquisition, however, the levels of methylation decreased noticeably. The third pattern (4/48 sites) showed potential incomplete erasure in 13.5 dpc primordial germ cells retaining higher than average levels of methylation followed by increasing methylation at subsequent time points (Fig. 3C). Finally, several sites (5/48) retained low levels throughout germ line development completing spermatogenesis with less than 20% methylation (Fig. 3D). A representative site was chosen for each of the first three patterns and bisulfite sequencing was used to confirm the qAMP results (Fig. 3E–G).

Previous studies have considered a number of genomic elements that may be responsible for guiding the site-specific acquisition of DNA methylation including CpG periodicity [39], small RNAs [40], and proximity to a gene involved in germ cell development [20]. To date, no satisfactory explanation has been uncovered to explain the directed nature of DNA methylation acquisition during male germline development. Chromosome 9 sites as well as the flanking 1 Kb, 5 Kb, and 10 Kb regions were grouped according to pattern type and genomic elements with the potential to direct DNA methylation were examined using a bioinformatic approach. No relationship was found between pattern type and the level of conservation or the proximity of repeat elements, scaffold matrix attachment regions (S/MARS), known genes, expressed transcripts, and miRNAs. Distance to the nearest transcription start site also did not appear to associate with pattern type.

### Time-dependent DNA methylation defects in the absence of DNMT3L in prenatal germ cells

**Figure 4:**
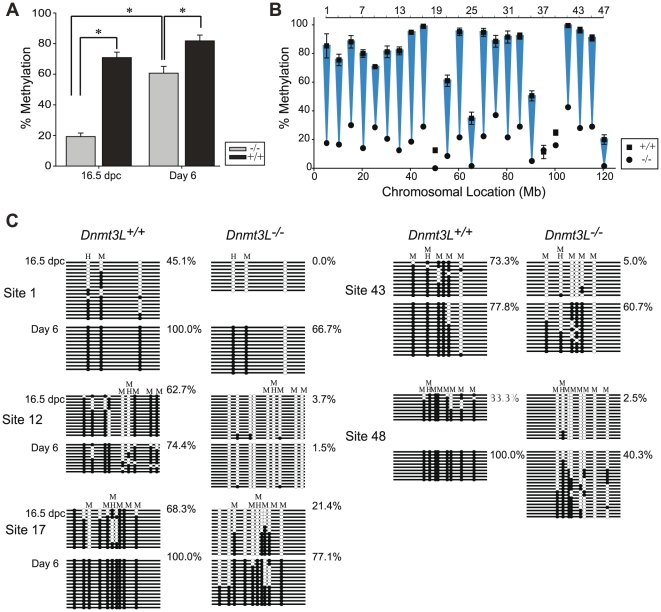
Analysis of DNA methylation in 16.5 dpc and day 6 male germ cells from *Dnmt3L^+/+^* and *Dnmt3L^−/−^* mice demonstrating a wide-spread loss of methylation across chromosome 9. **A**) Average percent methylation across chromosome 9 at 16.5 dpc and day 6 in *Dnmt3L^+/+^* (n = 70, n = 72) and *Dnmt3L^−/−^* (n = 24, n = 48) germ cells. Error bars indicate SEM across the chromosome. Significance was determined using the Mann-Whitney test of individual measurements across the chromosome (* indicates p<0.001). **B**) Comparison of *Dnmt3L^+/+^* (n = 3) and *Dnmt3L^−/−^* (n = 1) germ cells at 16.5 dpc demonstrating a drastic loss of DNA methylation at most sites examined. Error bars indicate SEM. **C**) Bisulfite sequencing at sites 1, 12, 17, 43 and 48 in *Dnmt3L^+/+^* and *Dnmt3L^−/−^* germ cells at 16.5 dpc and day 6.

**Figure 5:**
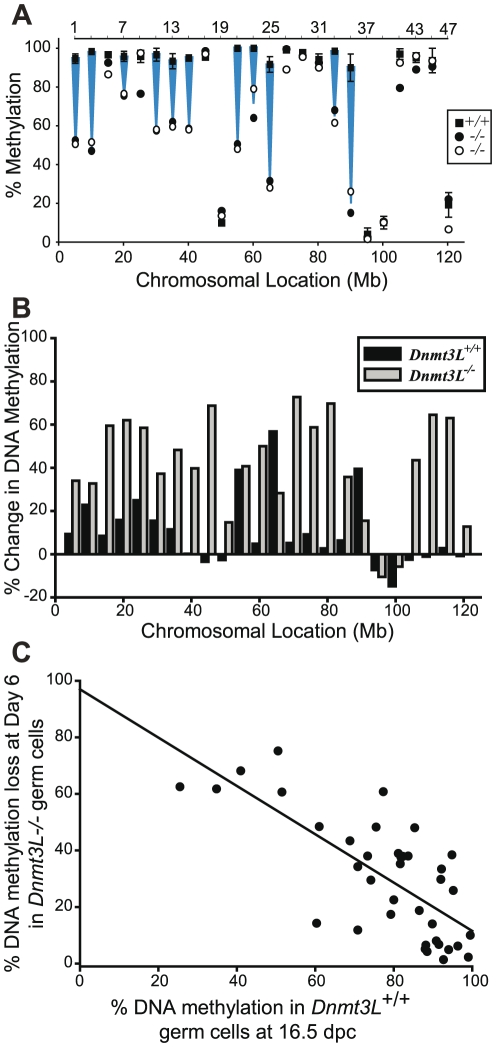
DNA methylation in male germ cells of wildtype and DNMT3L deficient mice. **A**) Comparison of *Dnmt3L^+/+^* (n = 3) and *Dnmt3L^−/−^* (n = 2) germ cells at day 6. Loss of DNA methylation was apparent at 11/24 sites in *Dnmt3L^−/−^* germ cells. Error bars indicate SEM. **B**) Percent change of DNA methylation from 16.5 dpc to day 6 in *Dnmt3L^+/+^* and *Dnmt3L^−/−^* cells. **C**) Linear regression analysis of the level of DNA methylation present in *Dnmt3L^+/+^* 16.5 dpc prospermatogonia and the percent loss of DNA methylation in *Dnmt3L^−/−^* day 6 spermatogonia at sites that obtain greater than 60% methylation during normal germ cell development (R = 0.732, n = 38, p<0.001). A linear regression analysis determined a significant (p<0.001) negative correlation (−0.732).

A loss of DNA methylation at paternally methylated imprinted genes *H19*, *Rasgrf1*, and *Dlk1/Gtl2* as well as at most classes of repetitive DNA elements has been shown in DNMT3L deficient male germ cells [1], [25], [31], [33]. Numerous intergenic loci were also shown to have decreased DNA methylation in *DnmtL^−/−^* type A spermatogonia at day 6 on chromosomes 4 and X [34]. In order to examine the prenatal effect of loss of DNMT3L and extend analysis to chromosome 9, germ cells were collected using FACS sorting at 16.5 dpc and day 6 from *Dnmt3L^+/+^* GFP^+^ and *Dnmt3L^−/−^* GFP^+^ male mice. DNA methylation across chromosome 9 at 5 Mb intervals was determined with qAMP analysis at the 24 odd numbered sites from the 48 sites examined above. These sites were chosen to accommodate the limited amount of sample available from *Dnmt3L^−/−^* mice as well as span the chromosome in an equidistant fashion. At 16.5 dpc, the loss of DNMT3L resulted in a nearly complete absence of DNA methylation in *Dnmt3L^−/−^* prospermatogonia (19.3%) compared to control *Dnmt3L^+/+^* prospermatogonia (70.9%) at sites examined on chromosome 9 (Fig. 4 A,B). Due to the challenge of obtaining a sufficient number of *Dnmt3L^−/−^* germ cells at 16.5 dpc for qAMP analysis, bisulfite sequencing at sites 1, 12, 17, 43 and 48 was used to confirm the severe loss of DNA methylation (Fig. 4C). By day 6, DNA methylation in *Dnmt3L^−/−^* spermatogonia had increased significantly (p<0.001) to 60.7% but was still significantly (p<0.001) different from *Dnmt3L^+/+^* spermatogonia (81.7%) (Figs. 4A, 5A). Decreased DNA methylation was present at 11/24 sites in *Dnmt3L^−/−^* spermatogonia (Fig. 5A). Nine out of 24 sites, however, attained normal DNA methylation levels in the *Dnmt3L^−/−^* spermatogonia. These results were also confirmed through bisulfite sequencing of sites 1, 12, 17, 43, and 48 (Fig. 4C). Examination of the percent change of DNA methylation from 16.5 dpc to day 6 showed an average increase of 41.4% in *Dnmt3L^−/−^* germ cells, which was significantly different (p<0.001) from the 10.1% average increase in *Dnmt3L^+/+^* germ cells (Fig. 5B) indicating that methylation acquisition may be delayed in the germ cells of DNMT3L deficient mice.

A linear regression analysis determined a significant (p<0.001) negative correlation (−0.732) between the level of DNA methylation present in *Dnmt3L^+/+^* 16.5 dpc prospermatogonia and the percent loss of DNA methylation in *Dnmt3L^−/−^* day 6 spermatogonia at sites that obtain greater than 60% methylation during normal germ cell development (Fig. 5C). This indicates that sites that normally acquire DNA methylation earlier in male germ cell development are less affected by the absence of DNMT3L in day 6 spermatogonia once again pointing towards a delay in DNA methylation acquisition in *Dnmt3L^−/−^* germ cells.

Although a number of sites examined attained normal levels of DNA methylation in DNMT3L deficient males, germ cell numbers were markedly reduced by day 6 [34]. To determine whether this loss commences during prenatal gametogenesis, testes from 18.5 dpc *Dnmt3L^+/−^* and *Dnmt3L^−/−^* embryos were collected and germ cell counts were obtained as previously described [34], [35]. Germ cell counts at 18.5 dpc revealed that loss of DNA methylation detected at 16.5 dpc did not significantly affect germ cell number (Fig. S4).

### *Dnmt* expression in DNMT3L deficient germ cells

**Figure 6:**
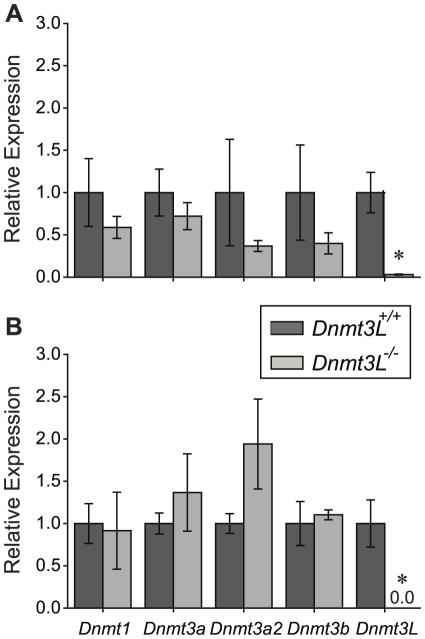
Expression analysis of *Dnmt1*, *Dnmt3a*, *Dnmt3a2*, *Dnmt3b* and *Dnmt3L* in *Dnmt3L^+/+^* and *Dnmt3L^−/−^* 16.5 dpc (A) and 18.5 dpc (B) prospermatogonia (n = 3 separate sets of germ cells per time point). Expression of each gene was normalized to 18S expression and is represented relative to the amount measured in *Dnmt3L^+/+^* germ cells for each time point for each gene. Asterisks denote a significant (t-test, p<0.05) change in DNA methylation compared to the *Dnmt3L^+/+^*.

From 16.5 dpc to day 6, DNA methylation across chromosome 9 increased significantly more (p<0.001) in *Dnmt3L^−/−^* germ cells as compared to *Dnmt3L^+/+^* germ cells (Fig. 5B) indicating either a delay in DNA methylation or a mechanism of compensation potentially mediated by the DNMT enzymes. DNMT3L deficient oocytes have been found to have an up-regulation of *Dnmt3a* and *Dnmt3b* transcripts at day 15, a major period during oocyte DNA methylation acquisition [41], [42]. The same phenomenon was not observed in *Dnmt3L^−/−^* day 6 spermatogonia [34], which is after the main period of DNA methylation acquisition. Since the majority of DNA methylation appears to occur prenatally in male gametogenesis, *Dnmt* expression was examined in 16.5 dpc and 18.5 dpc FACS sorted germ cells from timed matings between *Dnmt3L^+/−^* GFP-positive males and females. QRT-PCR was performed to analyze the expression of *Dnmt1*, *Dnmt3a*, *Dnmt3a2*, *Dnmt3b*, and *Dnmt3L* in *Dnmt3L^+/+^* and *Dnmt3L^−/−^* germ cells. As expected, *Dnmt3L* expression was absent in the *Dnmt3L^−/−^* testes at both 16.5 dpc and 18.5 dpc. Examination of the DNMT linked predominantly to maintenance DNA methylation, *Dnmt1*, and those linked with *de novo* DNA methylation, *Dnmt3a*, *Dnmt3a2* and *Dnmt3b* revealed no evidence of compensation for the absence of DNMT3L. No significant change of expression was found at either 16.5 dpc or 18.5 dpc (Fig. 6).

## Discussion

Accurate establishment of DNA methylation patterns is critical to the development of male germ cells and fertility. The timing of methylation erasure and acquisition at imprinted genes, repetitive elements, and a small handful of unique sequences has been reported. Recent genome-wide approaches, including next generation sequencing, in mouse and human have demonstrated that DNA methylation is frequently found in regions outside of proximal promoters, including intergenic sequences [16], [17]. DNA methylation at intergenic sequences may play roles in regulating gene expression and/or chromosome compaction during cell division and meiosis. Eckhardt et al [18] determined that evolutionarily conserved non-protein coding regions that were also tissue differentially methylated regions, were located up to 100 kb from the nearest annotated gene, consistent with potential long-range regulatory elements such as silencers or enhancers. Additionally, *Dnmt3L^−/−^* spermatocytes have been found to have heterochromatin marker abnormalities indicating a more open DNA conformation and poor chromatin packaging prior to entry into meiosis [33]. This evidence suggests that DNA methylation in the germline at intergenic regions may play a role in germline development. Due to the difficulty in isolating sufficient numbers of pure germ cells, the extent and precise timing of DNA methylation during the prenatal period is currently unclear for most of the estimated 20 million sites where DNA methylation occurs. In this paper, we determined the dynamics of prenatal DNA methylation at intergenic sequences and their perturbation in the absence of DNMT3L.

During the period of erasure in the PGCs, the majority of intergenic sites examined were depleted of DNA methylation by 13.5 dpc. Notably, along chromosome 9, 4/48 sites demonstrated incomplete erasure (25–60% methylation) indicating that DNA methylation may be retained from the parental patterns and suggesting a potential for transgenerational epigenetic inheritance at intergenic sites. This information combined with previous studies that observed retention of DNA methylation at repetitive elements [7] in the male germline reveals that incomplete erasure in PGCs may be more common than previously thought. While in most cases, partial retention of parental methylation patterns appears to be innocuous, deleterious epigenetic errors could also be transmitted between generations as has been recently described in the agouti viable yellow mouse model [43]. The full impact of these inherited patterns would depend on the extent of the epigenetic “resetting” that occurs during preimplantation embryonic development which has not been closely examined.

Examination of intergenic sites on chromosome 9 at 16.5 dpc showed a dramatic increase of DNA methylation compared to that at 13.5 dpc. This finding fits with studies of imprinted genes and repetitive elements that show methylation acquisition in the same prenatal window. Additionally, cooperating *de novo* DNA methyltransferases, *Dnmt3a2* and *Dnmt3L* are both highly expressed during this time period in prenatal germ cells [44], [45], [46] supporting the idea that the majority of male germline genomic DNA methylation acquisition occurs *in utero*. Following acquisition, in contrast to the majority of sites examined on chromosome 9, 6/48 sites consistently became demethylated to achieve their final level of DNA methylation. These dynamic modifications may be necessary to allow developing germ cells to transition through the early stages of gametogenesis or for meiosis.

DNMT3L deficient male germ cells have previously been collected from postnatal mice and shown to have a loss of DNA methylation at imprinted genes, repetitive elements, and some intergenic regions [1], [31], [33], [34]. Similarly, in this study chromosome 9 demonstrated a significant loss of DNA methylation in *Dnmt3L^−/−^* spermatogonia 6 days after birth (60.7% in *Dnmt3L^−/−^*versus 81.7% in *Dnmt3L^+/+^*). DNA methylation at intergenic sites in prenatal prospermatogonia from DNMT3L deficient mice were examined for the first time here and found to be severely hypomethylated along chromosome 9 in *Dnmt3L^−/−^* (19.3%) germ cells compared to *Dnmt3L^+/+^* (70.9%) germ cells. Interestingly, between 16.5 dpc and day 6, DNA methylation became fully established at a number of sequences (9/24) in the *Dnmt3L^−/−^* germ cells, especially those that are normally methylated earlier in germ cell development. The results indicate a delay in the timing of methylation acquisition in prenatal male germ cells lacking DNMT3L as compared to those from wild-type mice. It is possible that sequences that normally get methylated later during prenatal male germ cell development may be more susceptible to perturbation in DNMT levels, availability of methyl donors or perhaps exposure to adverse prenatal environments. To our knowledge, time-dependent aspects of DNA methylation acquisition have to date only been described in detail for imprinted genes. In the male germline, the paternal alleles of *H19, Rasgrf1 and Dlk1/Gtl2* become methylated before the maternal allele [1], [11], [12] and in the maternal germline a similar allele-specific timing of methylation occurs for *Snrpn, Zac1 and Peg1/Mest* [42], [47]. Furthermore, during the oocyte growth phase when maternal methylation imprints are acquired, some imprinted genes are methylated earlier than others [42], [47].

Examination of nonpromoter DNA methylation during both normal and DNMT3L deficient gametogenesis illustrated that acquisition occurs in a time and site-dependent manner. Sequence characteristics such as spacing between CpG dinucleotides, CpG flanking sequences, and organization of repetitive sequences have all been implicated in the determination of the location and levels of DNA methylation [48], [49]. No definitive mechanism for the targeting of DNMT3L-mediated DNA methylation to specific genomic regions has been determined. The sites examined in this study each demonstrated one of four patterns of DNA methylation acquisition as well as were either consistently demethylated or methylated in the absence of DNMT3L. Despite this evidence that DNMT3L-mediated DNA methylation occurs in a non-random fashion, bioinformatic analysis of the level of conservation and the proximity of repeat elements, S/MARS, known genes, expressed transcripts, and miRNAs within 1 Kb, 5 Kb, and 10 Kb flanking the sites examined revealed no common distinguishing factors. Distance to the nearest transcription start site also did not appear to associate with the pattern type of a site. A number of studies have implicated chromatin modifications in controlling the accessibility of genomic loci to DNA methylation enzymes. For instance, in a study by Ooi et al [50], it was determined that DNMT3L specifically interacted with histone 3 when it was unmethylated at lysine 4 (H3K4). Methylation of H3K4 strongly inhibited this interaction indicating a mechanism for controlling the sites at which DNMT3L-mediated DNA methylation might occur [51], [52]. At this time, data is not available for the methylation status of H3K4 within the critical window of DNA methylation during male germ cell development. Further studies including establishing the underlying chromatin structure and determining which small RNAs are present in gonocytes are warranted to fully understand DNA methylation pattern dynamics as well as mechanisms underlying DNMT3L function in prospermatogonia.

Although we could not identify sequence elements or context that would predict methylation patterning of intergenic sequences in the male germline, we did observe differences in methylation between individual CpGs within a given region. During normal development, differences in methylation between individual CpGs of intergenic sequences, as determined by examining our bisulfite sequencing results, were most frequently seen in 16.5 dpc cells and were resolved (i.e. uniform across the region) by postnatal day 6. Similar findings have been reported by others for imprinted genes. Both the paternal and maternal alleles of *H19* showed methylation differences between individual CpGs at 16.5 dpc; the differences were still present at 18.5 dpc but were resolved by postnatal day 0 [1]. We suggest that differences between sites during the early phase of *de novo* methylation of DNA in male germ cells reflects the normal methylation acquisition dynamics catalyzed by DNMT3a and amplified by DNMT3L. The fact that differences in methylation between CpGs in a specific intergenic region persisted at day 6 in the absence of DNMT3L may provide further insight into the role of DNMT3L in male germ cells. Recent studies on human DNMT3L have provided data suggesting that DNMT3L promotes more uniform methylation patterns in part by stimulating methylation of sites that are least methylated by DNMT3A [53]. Our data on germ cells from *Dnmt3L^−/−^* mice support the latter findings, in that differences in methylation between CpG sites (lack of uniformity) persisted at day 6 and some sites were the least methylated at 16.5 dpc, perhaps those that were less strongly methylated by DNMT3a, were most affected by the loss of DNMT3L. Thus both developmental timing and expression of the DNMT3s involved in *de novo* methylation may be critical for male germ cell DNA methylation patterning associated with normal fertility.

## Materials and Methods

### Mice

CD-1 mice were purchased from Charles River Canada Inc. (St-Constant, QC, Canada). Mice with mutated Dnmt3L (*Dnmt3L^tm1Bes^*, a gift from Timothy Bestor and Deborah Bourc'his) [31] and GOF/deltaPE-Oct4/GFP transgenic mice (a gift from Hans Scholer) [38] have been described elsewhere. *Dnmt3L^+/−^* females were crossed with GOF/deltaPE-Oct4/GFP males to obtain *Dnmt3L^+/^* ^−^, GFP^+^ mice. Noon of the day on which the vaginal plug was identified was considered 0.5 days post coitum (or dpc), while the day of birth was designated postpartum day (or dpp) 0. All procedures were carried out in accordance with the Canadian Council on Animal Care and approved by the McGill University Animal Care Ethics Committee with the protocol number of 3595.

### Isolation of Male Germ Cells by Flow Cytometry

Testes were collected from male embryos at 12.5 dpc, 13.5 dpc, 16.5 dpc, and 18.5 dpc and male pups at day 6 resulting from timed-pregnancies between CD-1 females and GOF/deltaPE-Oct4/GFP males. GFP^+^ paired testes with the three possible *Dnmt3L* genotypes were collected from male embryos at 16.5 dpc and male pups at day 6 from timed-pregnancies between *Dnmt3L^+/−^*,GFP^+^ males and females. Decapsulated testes were digested in 0.25% trypsin-EDTA (Gibco-BRL/Invitrogen, Burlington, ON, Canada) for 10 minutes at 37°C, dispersed and digested for another 10 minutes. DNase was added for the final 5 minutes of digestion. The resulting cell suspension was washed twice and resuspended in sterile phosphate buffer solution (PBS) with DNase. GFP-positive prospermatogonia and primitive type A spermatogonia were collected by MoFlow cell sorter (Cytomation Inc., Ft. Collins, CO).

### DNA Methylation Analysis

#### qAMP

DNA methylation analysis of germ cells and liver tissue was performed as described [36]. Briefly, isolated DNA was digested with no enzyme (sham), a methylation-sensitive restriction enzyme (HhaI), or a methylation-dependent restriction enzyme (McrBC). Genomic regions containing restriction enzyme cut sites were selected at intergenic locations defined as no gene or CpG island within 10 Kb on either flank as well as containing no repeat elements at 2.5 Mb intervals along chromosome 9 using the mm8 build of the UCSC Genome Browser (http://genome.ucsc.edu/) and PCR primers flanking the cut sites were designed (Table S1). Real-time PCR was performed on digested templates using QuantiTect™ SYBR® Green PCR kit (Qiagen Inc., Mississauga, ON, Canada) according to the manufacturer's suggestions for use with of the Mx3000P PCR machine (Stratagene, La Jolla, CA). The change in cycle threshold values relative to the sham digested template were utilized to determine the percent methylation for the amplified region. Results are expressed as an average of the percent methylation obtained from each enzymatic digestion unless otherwise noted.

#### Bisulfite Sequencing

DNA was isolated from 12.5 dpc, 13.5 dpc, 16.5 dpc and day 6 FACS sorted male germ cells. Bisulfite treatment was carried out using the EpiTect Bisulfite Kit according to the manufacturer's recommendations (Qiagen). Nested or semi-nested primers were designed to amplify sites 1, 12, and 48 on Chromosome 9 (Table S2). Two rounds of PCR were completed for each site. Each 23 uL PCR reaction contained 1 uL bisulfite converted DNA, 0.5 uL of each primer (10 uM), 12.5 uL PCR Mastermix (Promega Corporation), and 8.5 uL nuclease free water. The following PCR conditions were used: 2 minutes at 94°C, 1 minute at 53°C, 1 minute at 72°C for two cycles followed by 30 seconds at 94°C, 1 minute at 53°C, 1 minute at 72°C for 40 cycles and 1 cycle of 10 minutes at 72°C. The second round of PCR was carried out in the same manner with 5 uL PCR product from the first round of PCR. PCR products were separated on a 2% agarose gel and extracted using the Qiagen MiniElute Gel Extraction Kit (Qiagen). PCR products were subcloned using the TOPO TA Cloning Kit (Invitrogen) and plasmid DNA was purified with the QIAprep Spin Miniprep Kit (Qiagen). Ten to twenty clones were selected per region and sent for sequencing at Genome Quebec. Figures were created with the assistance of QUantification tool for Methylation Analysis (QUMA) (http://quma.cdb.riken.jp/) [54].

### Bioinformatic Analysis

Chromosome 9 sites were examined using bioinformatic tools available through the UCSC Genome Browser (mm8). Custom tracks of the chromosome 9 sites were created to include only the sites as well as individual tracks including an additional 1 Kb, 5 Kb, and 10 Kb in either direction of each site. The Table Browser feature of the UCSC Genome Browser was then used to intersect these tracks with tracks available on the UCSC browser of known genes, expression (GNF Atlas 2), miRNA, conservation, and repeat elements (including segmental duplications, RepeatMasker, simple repeats and microsatellites). In addition, a custom track of transcription start sites was created from database version 6 available through DataBase of Transcriptional Start Sites (www.dbtss.hgc.jp) and intersected with the chromosome 9 custom tracks mentioned above. Finally, the presence and proximity of scaffold matrix attachment regions (S/MARS) was analyzed using SMARTest from Genomatix (http://www.genomatix.de/) for each of the chromosome sites with and without an additional flanking 1 Kb, 5 Kb, and 10 Kb.

### Quantitative RT-PCR

Total RNA was extracted from 16.5 dpc and 18.5 dpc FACS sorted male germ cells from timed-pregnancies between *Dnmt3L^+/−^*,GFP^+^ males and females using the PicoPure RNA Isolation Kit (Molecular Devices, Sunnyvale, CA, USA). Total RNA was reverse transcribed using Moloney Murine Leukemia Virus (M-MLV) reverse transcriptase (Invitrogen) using both random hexamers and oligo-dTs as primers. Quantitative real-time PCR (qRT-PCR) was performed using the QuantiTect™ SYBR® Green PCR kit (Qiagen) according to the manufacturer's suggestions for use with of the Mx3000P PCR machine (Stratagene). The primers and conditions used to determine the expression of *Dnmt1*, *Dnmt3a*, *Dnmt3a2*, *Dnmt3b*, and *Dnmt3L* each normalized to 18S expression, according to the standard curve method, have been described previously [44], [55], [56]. Reactions were done in triplicate using three individual sets of germ cells for each genotype at each time point.

### Germ Cell Counts

*Dnmt3L^+/−^* and *Dnmt3L^−/−^* testes were obtained from 18.5 dpc male embryos by crossing *Dnmt3L^+/−^* mice. Testes were immersed in Bouin's fixative (BDH Inc., Toronto, ON, Canada) for 4 hours, dehydrated, and embedded in paraffin. Testes were cut into 5 um serial sections with every fifth section used for germ cell quantification. Germ cells were counted by an individual blinded to the slide identity and are reported per 2000 Sertoli cells as described [34], [35], [57]. Germ cells from three individual animals were counted per genotype and at least four sections per animal were used for counting. A Zeiss AxioImager Z1 microscope was used to view slides.

### Statistical Analysis

Data were analyzed with the aid of SigmaPlot computer program (SigmaPlot v11.0, Systat Software Inc, San Jose, CA, USA). The DNA methylation data obtained using qAMP was analyzed using the Mann-Whitney rank sum test or *t*-test as indicated in the text and figure legends. In all cases, a P≤0.05 was considered significant. Data are presented as mean ±SEM. A linear regression analysis was used to relate the level of DNA methylation present in *Dnmt3L^+/+^* 16.5 dpc prospermatogonia and the percent loss of DNA methylation in *Dnmt3L^−/−^* day 6 spermatogonia. Only sites that obtained greater than 60% methylation during normal germ cell development were used for this analysis.

## Supporting Information

Figure S1 **DNA methylation of chromosome 4 at 13.5 dpc.** qAMP analysis of DNA methylation along chromosome 4 in male germ cells from 13.5 dpc embryos (only HhaI digests are shown, n = 1) show a similar pattern of DNA methylation erasure as on chromosome 9. Red circles indicate no data available. **Supplemental methods for Figure 1.** DNA methylation analysis of germ cells and liver tissue was performed as described [36]. Briefly, isolated DNA was digested with no enzyme (sham) as well as a methylation-sensitive restriction enzyme (HhaI). Primers flanking intergenic regions containing restriction enzyme cut sites at 5 Mb intervals along chromosome 4 were used [15]. Real-time PCR was performed on digested templates using QuantiTect™ SYBR® Green PCR kit (Qiagen Inc., Mississauga, ON, Canada) according to the manufacturer's suggestions for use with of the Mx3000P PCR machine (Stratagene, La Jolla, CA). The change in cycle threshold values relative to the sham digested template were utilized to determine the percent methylation for the amplified region..
(TIF)

Figure S2 **Expression dynamics of DNA methyltransferases in prenatal male germ cells.** Relative quantification of **A**) *Dnmt3L*, **B**) *Dnmt3a*, **C**) *Dnmt3b* and **D**) *Dnmt1* expression in purified populations of male germ cells. Quantitative RT-PCR was used to determine the global expression levels of these genes in total RNA extracted from E13.5, E15.5 and E18.5 prospermatogonia and 6 dpp primitive type A spermatogonia (PA). Note the difference in scale magnitude between A) and B), C), D). Expression of each gene was determined in triplicate in each of the two series of germ cells and normalized to 18S expression; normalized values were calibrated to the expression found in E13.5 gonocytes. Shown here are the mean expression results obtained for one series. Mean ± SD.
(TIF)

Figure S3 **Differential expressions of Dnmt3a and Dnmt3a2 in prenatal male germ cells.** Relative expression of Dnmt3a (top) and Dnmt3a2 (bottom) in purified populations of male germ cells. Real-time RT-PCR was used to determine the expression levels of the two transcripts in total RNA extracted from E13.5, E15.5 and E18.5 prospermatogonia and 6 dpp primitive type A spermatogonia (PA). Expression of each transcript was determined in triplicate in each of the two series of germ cells and normalized to 18S expression; normalized values were calibrated to the expression found in E13.5 gonocytes. Shown here are the mean expression results obtained for one series. Mean ± SD. **Methods for Figures S2 and S3.** Total RNA was extracted from snap-frozen pellets of male germ cells using the RNeasy Mini kit with DNaseI treatment according to the manufacturer's protocol (Qiagen Inc., Mississauga, ON, Canada). Real-Time or quantitative RT-PCR (qRT-PCR) was performed using the Mx4000 qPCR system from Stratagene (La Jolla, CA) using the QuantiTect™ SYBR® Green RT-PCR kit (Qiagen) as described previously [44]. The primers used to determine the overall relative expression levels of *Dnmt1*, *Dnmt3a*, *Dnmt3b* and *Dnmt3L* according to the standard curve method have been described elsewhere [44], [56]. Note that the primers assaying *Dnmt3L* expression were designed to pick up the prospermatogonia (full-length) form of Dnmt3L and not any of the other spermatid-specific transcript variants described [58]. The transcript-specific primers designed to assess Dnmt3a and Dnmt3a2 expression were the same as in [55]. In all cases, reactions were performed in triplicate on the same two independent sets of germ cells. Expression results were normalized to their corresponding 18S rRNA content. Fold changes in expression for a given gene were determined in relation to the expression of that gene in E13.5 gonocytes for prenatal germ cells or in pachytene spermatocytes for postnatal germ cells; all other quantities are expressed as *n*-fold differences relative to the expression of that gene in these respective cells types. Representative data for one set of germ cells are presented as mean ± SD.
(TIF)

Figure S4 **Germ cell counts of *Dnmt3L^+/−^* and *Dnmt3L^−/−^* testes stained with H&E indicate no significant decrease in germ cell counts at 18.5 dpc (n = 3).** Error bars indicate standard error.
(TIF)

Table S1 **qAMP Primers: Primers were selected to amplify nonpromoter, intergenic, non-CpG and non-repetitive regions spanning chromosome 9 at 2.5 Mb intervals.**
(DOC)

Table S2 **Bisulfite primers.**
(DOC)
