# Supplementary figures and images for: Critical Period of Nonpromoter DNA Methylation Acquisition during Prenatal Male Germ Cell Development
Source: PLoS One. 2011 Sep 19;6(9):e24156. doi: 10.1371/journal.pone.0024156 (PMC3176233; doi:10.1371/journal.pone.0024156)

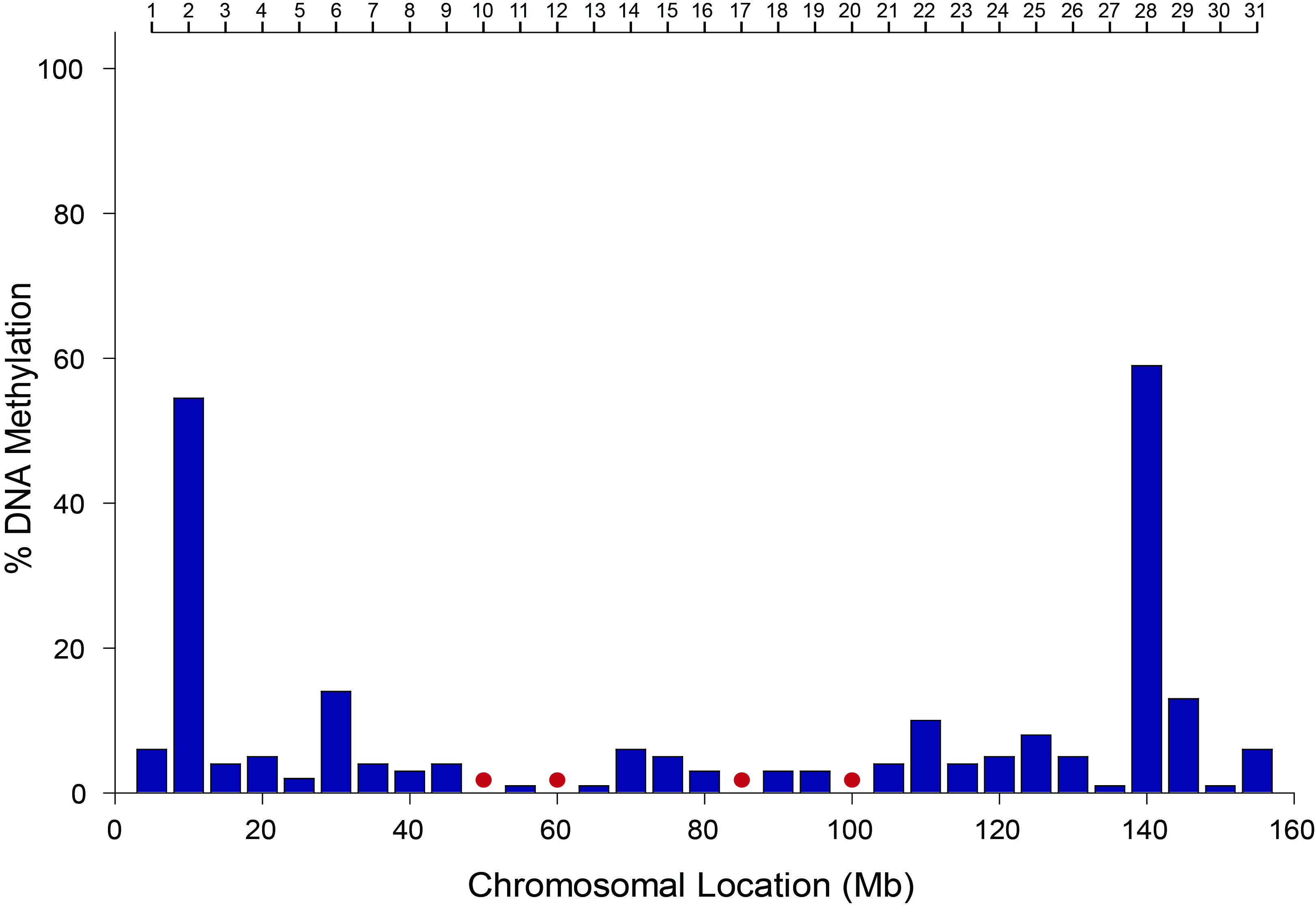

Supplement: Figure S1 — DNA methylation of chromosome 4 at 13.5 dpc. qAMP analysis of DNA methylation along chromosome 4 in male germ cells from 13.5 dpc embryos (only HhaI digests are shown, n = 1) show a similar pattern of DNA methylation erasure as on chromosome 9. Red circles indicate no data available. Supplemental methods for Figure 1. DNA methylation analysis of germ cells and liver tissue was performed as described [36]. Briefly, isolated DNA was digested with no enzyme (sham) as well as a methylation-sensitive restriction enzyme (HhaI). Primers flanking intergenic regions containing restriction enzyme cut sites at 5 Mb intervals along chromosome 4 were used [15]. Real-time PCR was performed on digested templates using QuantiTect™ SYBR® Green PCR kit (Qiagen Inc., Mississauga, ON, Canada) according to the manufacturer's suggestions for use with of the Mx3000P PCR machine (Stratagene, La Jolla, CA). The change in cycle threshold values relative to the sham digested template were utilized to determine the percent methylation for the amplified region.. (TIF) [file pone.0024156.s001.tif]

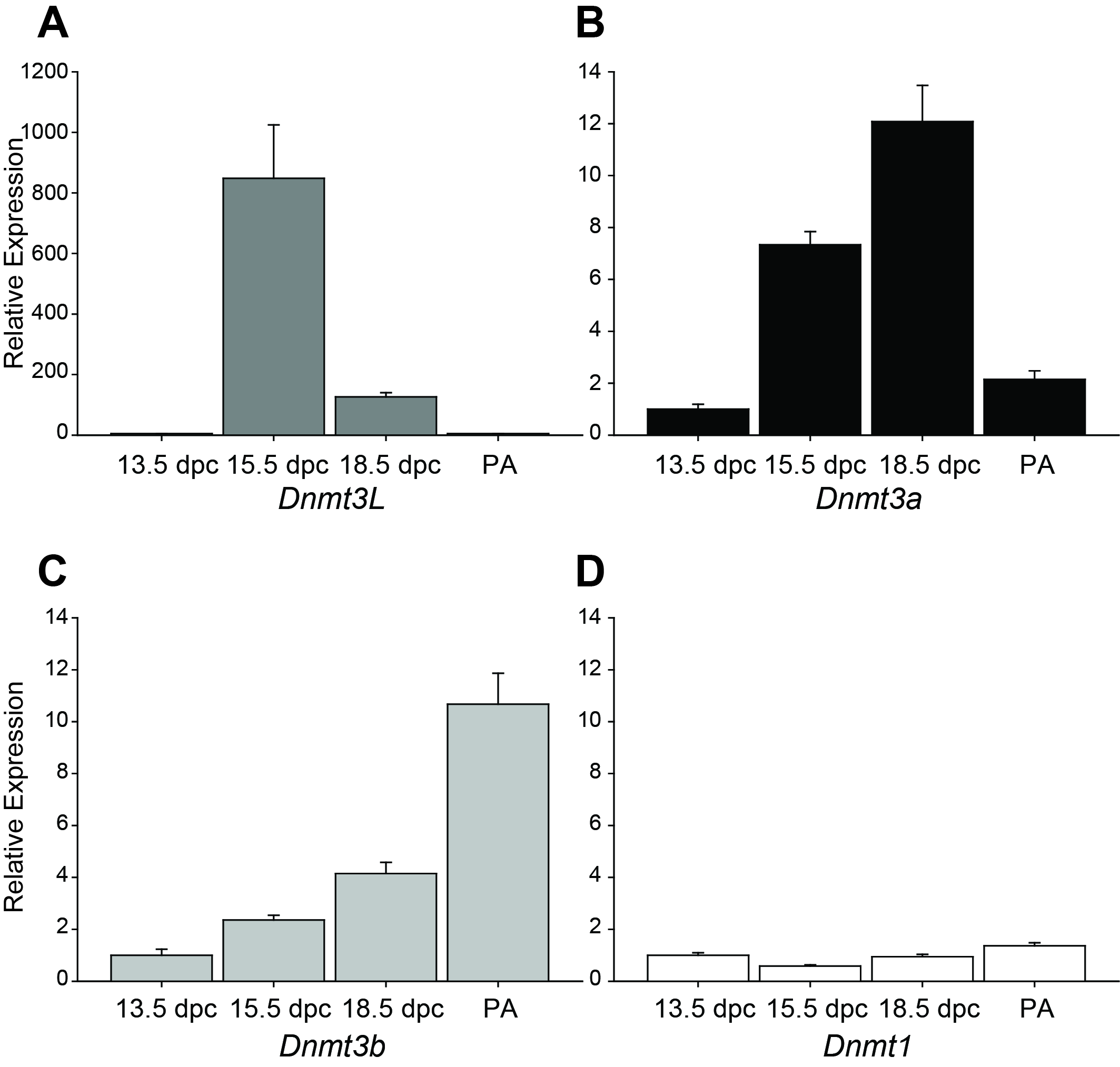

Supplement: Figure S2 — Expression dynamics of DNA methyltransferases in prenatal male germ cells. Relative quantification of A) Dnmt3L, B) Dnmt3a, C) Dnmt3b and D) Dnmt1 expression in purified populations of male germ cells. Quantitative RT-PCR was used to determine the global expression levels of these genes in total RNA extracted from E13.5, E15.5 and E18.5 prospermatogonia and 6 dpp primitive type A spermatogonia (PA). Note the difference in scale magnitude between A) and B), C), D). Expression of each gene was determined in triplicate in each of the two series of germ cells and normalized to 18S expression; normalized values were calibrated to the expression found in E13.5 gonocytes. Shown here are the mean expression results obtained for one series. Mean ± SD. (TIF) [file pone.0024156.s002.tif]

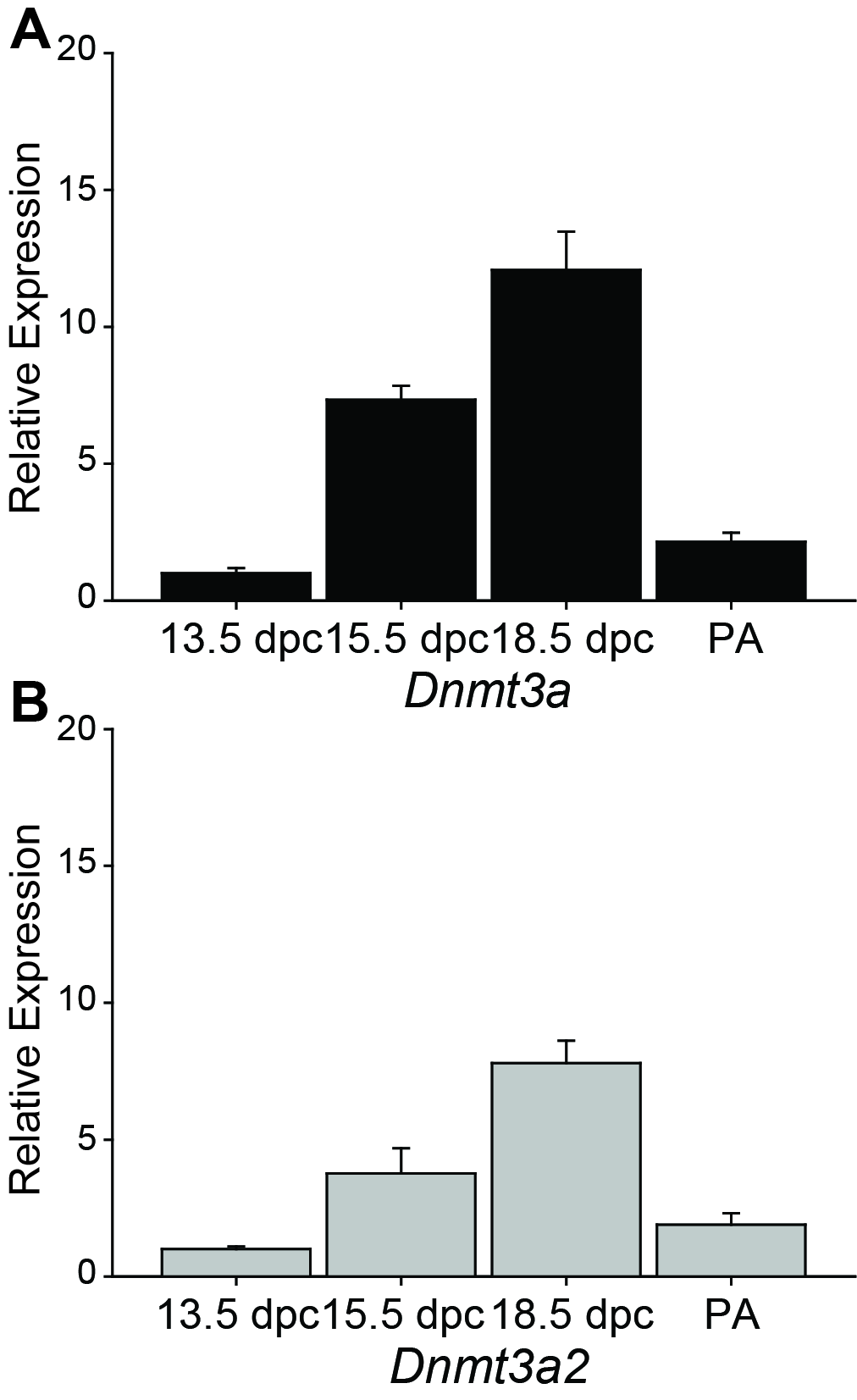

Supplement: Figure S3 — Differential expressions of Dnmt3a and Dnmt3a2 in prenatal male germ cells. Relative expression of Dnmt3a (top) and Dnmt3a2 (bottom) in purified populations of male germ cells. Real-time RT-PCR was used to determine the expression levels of the two transcripts in total RNA extracted from E13.5, E15.5 and E18.5 prospermatogonia and 6 dpp primitive type A spermatogonia (PA). Expression of each transcript was determined in triplicate in each of the two series of germ cells and normalized to 18S expression; normalized values were calibrated to the expression found in E13.5 gonocytes. Shown here are the mean expression results obtained for one series. Mean ± SD. Methods for Figures S2 and S3. Total RNA was extracted from snap-frozen pellets of male germ cells using the RNeasy Mini kit with DNaseI treatment according to the manufacturer's protocol (Qiagen Inc., Mississauga, ON, Canada). Real-Time or quantitative RT-PCR (qRT-PCR) was performed using the Mx4000 qPCR system from Stratagene (La Jolla, CA) using the QuantiTect™ SYBR® Green RT-PCR kit (Qiagen) as described previously [44]. The primers used to determine the overall relative expression levels of Dnmt1, Dnmt3a, Dnmt3b and Dnmt3L according to the standard curve method have been described elsewhere [44], [56]. Note that the primers assaying Dnmt3L expression were designed to pick up the prospermatogonia (full-length) form of Dnmt3L and not any of the other spermatid-specific transcript variants described [58]. The transcript-specific primers designed to assess Dnmt3a and Dnmt3a2 expression were the same as in [55]. In all cases, reactions were performed in triplicate on the same two independent sets of germ cells. Expression results were normalized to their corresponding 18S rRNA content. Fold changes in expression for a given gene were determined in relation to the expression of that gene in E13.5 gonocytes for prenatal germ cells or in pachytene spermatocytes for postnatal germ cells; all other quantities are expr [file pone.0024156.s003.tif]

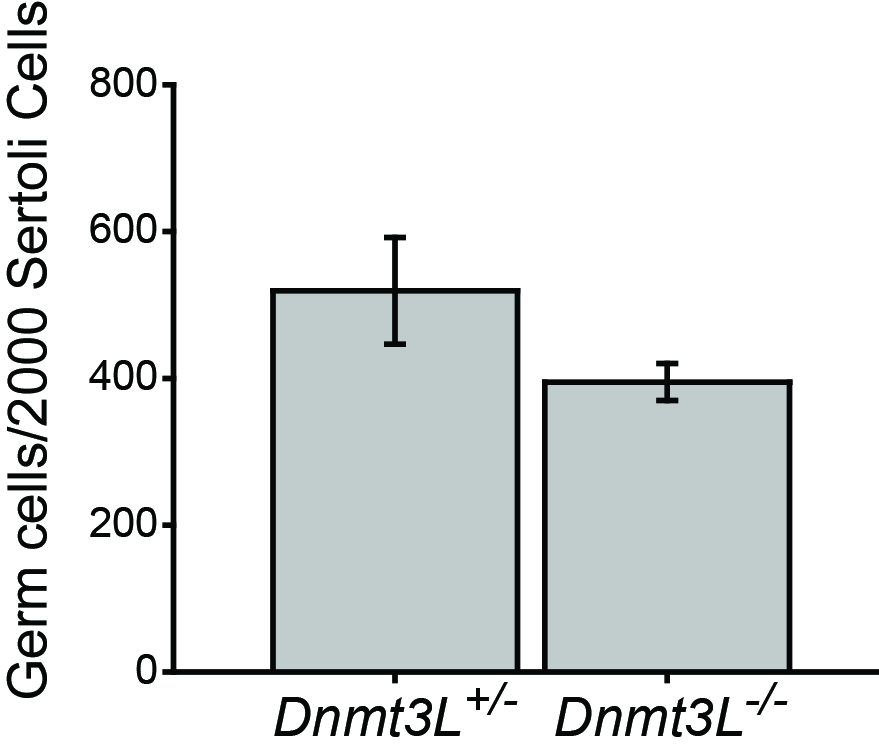

Supplement: Figure S4 — Germ cell counts of Dnmt3L+/− and Dnmt3L−/− testes stained with H&E indicate no significant decrease in germ cell counts at 18.5 dpc (n = 3). Error bars indicate standard error. (TIF) [file pone.0024156.s004.tif]
