# Supplementary material for: Critical Period of Nonpromoter DNA Methylation Acquisition during Prenatal Male Germ Cell Development
Source: PLoS One. 2011 Sep 19;6(9):e24156. doi: 10.1371/journal.pone.0024156 (PMC3176233; doi:10.1371/journal.pone.0024156)
Supplement: Table S1 — qAMP Primers: Primers were selected to amplify nonpromoter, intergenic, non-CpG and non-repetitive regions spanning chromosome 9 at 2.5 Mb intervals. (DOC) [file pone.0024156.s005.doc]

**Supplementary Table 1: qAMP Primers: Primers were selected to amplify nonpromoter, intergenic, non-CpG and non-repetitive regions spanning chromosome 9 at 2.5 Mb intervals.**

| **Region (mm8)** | **5' Primer** | **3' Primer** |
| --- | --- | --- |
| Chr9:5468894 | AGCATGACTGAGTCTCCCATCT | GAGCATTTTCCCCATTTTTC |
| Chr9:7914811 | AGCTGCACAAGGTAAGGATCA | TGGGGAATCCTGTGCACTAC |
| Chr9:10070078 | TCTGTACTACTGAGCAAAAAGGTCA | TCCCATCATGCCTAACAACA |
| Chr9:12573346 | TTCCCCTGTCCTCTGAGTTT | GGGGATACCTCACTCCCACT |
| Chr9:15266947 | AGGTGGCATGGAAGGTGTT | GCCAACTGTCCAACACTGAC |
| Chr9:17535476 | TTTCCTGGTGCTGGTATTTT | AAGCGGGTAAAGGAGAACAA |
| Chr9:20597129 | AGTTCATAAAAACTCGGGTGGA | AATGGGCAGCCTTTTCAGAT |
| Chr9:22704776 | CCCTTATTCCCTCGACCTCT | GTCCAGCCGCACATGTATC |
| Chr9:25381185 | AAGCCCTTCCCACAGTGAA | CCAGACCAACACACAATGCT |
| Chr9:27528341 | GGCTGCAAGTAGAGCTCAGTG | GTAGGCATCCTGAGCAATGG |
| Chr9:30282514 | CTTCAGCTTTCGTTTCTTCCA | AATAGGGGTTCTTTCACTGGAT |
| Chr9:32983196 | CTGAAACCCAGCCTCACATC | CACAACGAAAACCCTTCTCAA |
| Chr9:35194242 | TCTATTAGGCCGGCAGTTGT | CTTCCTGTCCAGAAGGTGCT |
| Chr9:37609511 | GGAGAATCATGGTCTCTGCTTT | GGTGTGACAGGTAATGCTATCTCA |
| Chr9:40074868 | CCAGGTCTTGGGTACTCTCCT | TCCTGGTGTCCTGTTCCTCT |
| Chr9:43040671 | ACCACCACAGCGGAACAAT | AACGATGTGACTGGTGCAAG |
| Chr9:45219174 | CCCTGGTGTCGGATTTAATG | GAAGGGGGAGGGATAGACAA |
| Chr9:47627496 | CCTGGATAGCCACCAAGACT | GAAAAGCATCACAAAGCAGGA |
| Chr9:50204604 | CTTCTGCTGCTCCTGCCTA | ACAACCACAGGTTATGTGACAG |
| Chr9:52631910 | TTCCCACCTGACTGGTTTCT | GCTGGGTCTTCCATGTCAGT |
| Chr9:55180688 | TCAGAACTCACACTTGCTCCT | TTGGAGAGGCAAATCAAAGA |
| Chr9:57115490 | CTCCCCTGCATCATTCTTTG | ACAGGAGGAGGAATGTGTGC |
| Chr9:60145023 | TCCTGGGGAAGATGTTTATCA | AAGCACACACTAGGGCAAAAA |
| Chr9:62831935 | CTTGGAGGATCCAGGTCAAG | CCTGCTCCTTGGTGATGACT |
| Chr9:65046056 | ACAAAGCCTTCCCTGTGGT | GGCCTACTCTCTACTGAGTTCCA |
| Chr9:67458180 | TGTTCCTGGTGGAGGTCAG | TCACAGCTGCTATAGGCTTCC |
| Chr9:70170986 | GGTTTTTCTGCCCTTATTGC | CTATGGAGGGGGTGAAGGA |
| Chr9:72568791 | ACGCTGACTGTGAAGACAGG | CTGATGTGGAAACGGAGGTC |
| Chr9:75053976 | GGTCCTGCCTGGAAGATGT | TCTGCTGTGCATCAGTTGTG |
| Chr9:77527645 | GAAGAGTCAGCTTCTCCTCAGC | TAGGAAGCAGGACGAGCAAT |
| Chr9:80063493 | CTGCATACAGCCTCTGATGG | CCCAACAAGAAGCAAAGTGA |
| Chr9:82507035 | AAGAGACAGGTGCGGAAAGA | GCCCCTGAGCTAACAGAGAA |
| Chr9:85002734 | GCTTTGGAGTAACAATGTGCAA | TTTCTGTCTCAAGAAGGAGGAA |
| Chr9:87576710 | GACATGAGGAACCAAAGACCA | TTGAGGCTCATATCCCCATT |
| Chr9:90982415 | ATTAGCACAGGGTAGCAAGG | TTCTTCATGGAGTTCTGTAGCA |
| Chr9:92637655 | CCCAGCAGCCTAGAAAATGA | AAAGCTTAATGCCACAGACCA |
| Chr9:95614663 | ACTGAGCCCAGAAGGCAAGT | GGCAGAGACCTCTGGAACTG |
| Chr9:98038118 | AATACCCACAGCAGGACCAG | GCACATGGTGTGGGAGAAGT |
| Chr9:100429190 | CCACACCCACTCTCTCACCT | CACACATCCACTGCCAAGAC |
| Chr9:102567725 | ATGGGCAAAACCAAAAGGAG | TGGGAGCCTCTTTTATCTGC |
| Chr9:105230170 | GCCATTACCACTCCGATTCT | CGAAGCAAACTCTGTTGCTG |
| Chr9:107503180 | CTGCTTCCTGAAGACCAAGG | GATTGCTTTCAACCCAGTCC |
| Chr9:110190398 | CAGGATGAGGGGTGAGGTTA | TGCTACAGTTTATGAGACCACCA |
| Chr9:112594485 | GATGTGAGCAACCAGGTGAA | GTGGTCAGTGCAGAAGCAAA |
| Chr9:115028441 | CCTCCCTTCTCTGGGAACA | GGCCCTTCAGGGGTTACTCT |
| Chr9:118076105 | GGTGCACTTTAGAGGCCAAA | TCTGACTGCAACCTGCACA |
| Chr9:120032890 | CGGAATCAGAATCAGCCTGT | CCTAGTCGGGTTCATGGCTA |
| Chr9:122357013 | CTGTCTGCCGCATTCTGAG | CCACGGAGATGGTGTTAAGC |
