# Supplementary material for: Critical Period of Nonpromoter DNA Methylation Acquisition during Prenatal Male Germ Cell Development
Source: PLoS One. 2011 Sep 19;6(9):e24156. doi: 10.1371/journal.pone.0024156 (PMC3176233; doi:10.1371/journal.pone.0024156)
Supplement: Table S2 — Bisulfite primers. (DOC) [file pone.0024156.s006.doc]

**Supplementary Table 2:** Bisulfite Primers

| **Site** | **5' Primer** | **3' Primer** |
| --- | --- | --- |
| Site 1 Outside | TTTGTTTTTTGAAGTTGGTAGA | CAAAAAAAACAAATAAAAACTCC |
| Site 1 Inside | ATGAGTTAAGGTTTTAAGTATGATTG | AATTCTAACATAAAAACTTCTCAAAAA |
| Site 12 Outside | GTGGGGAAGATTGAAGTTAGAT | AAACCCTTTACAAAACAAATTCC |
| Site 12 Inside | GGAAATGGTTTTTGAGTGAATT | CCAAAACAAACACAATTCAAAT |
| Site 17 Outside | GATAGTTGGGGTGGTTTTTTT | ACCCTAAAATCTTTCCCCTTTA |
| Site 17 Inside | TGGGGTGGTTTTTTTTTTAGTA | ACCCTAAAATCTTTCCCCTTTA |
| Site 43 Outside | GGTTTTTTTTTGAATGTGTAAGG | AATAACCACACTCACCACACTC |
| Site 43 Inside | GGAATAATTTAGTTGGAGAGTTGG | AATAACCACACTCACCACACTC |
| Site 48 Outside | GGTTTGGAATGTATGAAGAGATAT | AATTAATCAACCAAAAAAAAACT |
| Site 48 Inside | TTAAGGAAGTTTTTGTAAGGAA | CTAACTACAACCTACACAAAACACA |
